# Linc00472 suppresses proliferation and promotes apoptosis through elevating PDCD4 expression by sponging miR-196a in colorectal cancer

**DOI:** 10.18632/aging.101488

**Published:** 2018-06-21

**Authors:** Yafei Ye, Shengnan Yang, Yanping Han, Jingjing Sun, Lijuan Xv, Lina Wu, Yongfeng Wang, Liang Ming

**Affiliations:** 1 Department of Clinical Laboratory, The First Affiliated Hospital of Zhengzhou University, Henan, Zhengzhou 450000, China

**Keywords:** colorectal cancer, Linc00472, miR-196a, programmed cell death 4

## Abstract

Long intergenic non-coding RNA Linc00472 has been considered as a tumor suppressor in some cancers. However, the function and mechanism of Linc00472 in colorectal cancer has not been well elucidated. In this study, we found that Linc00472 was down-regulated in colorectal cancer tissues and cells. Elevated Linc00472 expression suppressed proliferation and induced apoptosis in colorectal cancer cells. Moreover, Linc00472 acted as a competing endogenous RNA (ceRNA) of miR-196a to release programmed cell death 4 (PDCD4). Furthermore, miR-196a overexpression or PDCD4 knockdown reversed Linc00472-mediated proliferation inhibition and apoptosis induction in colorectal cancer cells. Ectopic Linc00472 expression hindered tumor growth *in vivo*. Our study demonstrated that Linc00472 suppressed proliferation and induced apoptosis through up-regulating PDCD4 by decoying miR-196a, which may be an effective therapeutic target for colorectal cancer.

## Introduction

Colorectal cancer (CRC) is the third most common malignant neoplasm and the fourth leading cause of cancer-related death worldwide [1]. Despite remarkable progress in early detection and treatment of CRC, the prognosis of patients with advanced stage is still poor [2,3]. Therefore, it is urgent to elucidate the molecular mechanism underlying CRC carcinogenesis to identify more effective diagnostic strategies and potential therapeutic targets.

Long non-coding RNAs (lncRNAs) are an emerging group of transcripts >200 nucleotides in length, lacking protein coding potential. They exert their functional roles through disparate mechanisms by acting as guides, scaffolds, decoys or tethers of other biological molecules [4,5]. Increasing evidence demonstrates that lncRNAs are involved in various cellular biological processes in cancers such as growth, differentiation, tumorigenesis, apoptosis, invasion and stem cell pluripotency [6–8]. Notably, multiple studies have indicated that dysregulated lncRNAs, including UCA1 [9], TUG1 [10], DANCR [11] and CRNDE [12] functioned as oncogenes in CRC through a competing endogenous RNAs (ceRNAs) mechanism. Nevertheless, the functional mechanisms of lncRNAs in CRC progression remains largely unknown. Linc00472, a long intergenic non-coding RNA located on chromosome 6q13, was reported to be down-regulated and act as a tumor suppressor in breast cancer [13]. Moreover, high expression of Linc00472 was associated with low grade tumors and early stage disease in epithelial ovarian cancer [14]. Recently, Zhang et al. [15] found that low-expression of Linc00472 existed in rectal adenocarcinoma, a type of CRC. However, the functional role and molecular mechanisms of Linc00472 in CRC development remain not well elucidated.

In this study, we discovered that Linc00472 was down-regulated in CRC tissues and cells. Moreover, Linc00472 suppressed proliferation and induced apoptosis in CRC cells. Mechanically, Linc00472 acted as a tumor suppressor in CRC by up-regulating PDCD4 via sponging miR-196a. Our study demonstrated that Linc00472 may be a potential therapeutic target for CRC.

## RESULTS

### Linc00472 expression was downregulated in colorectal cancer tissues and cells

**Figure 1:**
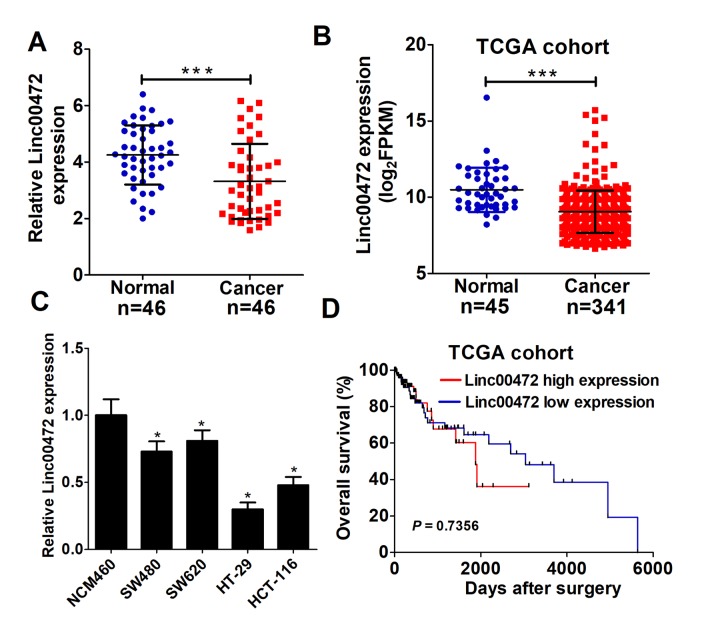
**Linc00472 was decreased in CRC tissues and cells.** (**A**) The expression levels of Linc00472 in CRC tumor tissues and adjacent normal tissues were determined by qRT-PCR assays. (**B**) Linc00472 expression was analyzed in TCGA colon adenocarcinoma (COAD) cohort. (**C**) qRT-PCR analysis was performed to detect Linc00472 expression in immortalized human colonic epithelial cell line NCM460 and four CRC cell lines (SW480, SW620, HT-29 and HCT-116). (**D**) Kaplan-Meier curve was employed to assess the overall survival outcome in CRC patients with high or low Linc00472 expression in TCGA dataset. *P < 0.05, ***P < 0.001.

To determine whether Linc00472 was dysregulated in CRC, qRT-PCR was performed to detect Linc00472 expression in 46 pairs of CRC tumor and adjacent non-tumor tissues. As showed in Figure 1A, Linc00472 levels was significantly down-regulated in tumor tissues compared with adjacent normal tissues, which was further verified by TCGA cohort analysis (Figure 1B). Moreover, the expression level of Linc00472 was distinctly decreased in CRC cells (SW480, SW620, HT-29 and HCT-116) in comparison with the immortalized human colonic epithelial cell line NCM460 (Figure 1C). A previous study reported that low Linc00472 expression led to poor prognosis in breast cancer [16]. Therefore, we further investigated its prognostic value in CRC using TCGA dataset. Kaplan–Meier survival analysis revealed that no significant difference existed between Linc00472 levels and overall survival of CRC (*P* = 0.7356, log-rank test) (Figure 1D), similar to another study on epithelial ovarian cancer [14]. All these data suggested that Linc00472 was down-regulated in CRC tissues and cells.

### Linc00472 overexpression suppressed proliferation and induced apoptosis in colorectal cancer cells

**Figure 2:**
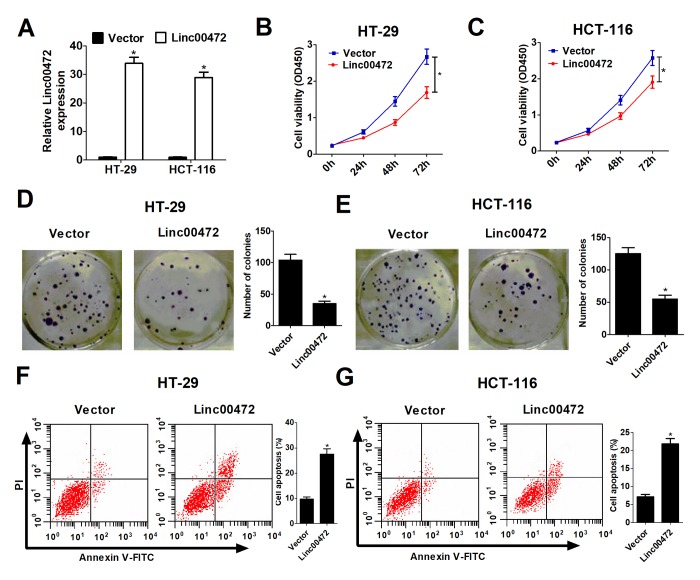
**Linc00472 overexpression inhibited proliferation and induced apoptosis in CRC cells.** HT-29 and HCT-116 cells were transfected with pcDNA3.1 vector (Vector) or pcDNA-Linc00472 (Linc00472). (**A**) The Linc00472 expression level was detected using qRT-PCR assays. (**B** and **C**) Cell proliferation was measured with CCK-8 assays. (**D** and **E**) Cell clone numbers were evaluated by colony formation assay. (**F** and **G**) Cell apoptosis was determined by flow cytometry analysis. *P < 0.05.

To explore the functional role of Linc00472 in CRC, Linc00472 overexpression vector (Linc00472) was transfected into HT-29 and HCT-116 cells. As expected in Figure 2A, Linc00472 transfecting HT-29 and HCT-116 cells had dramatically increased Linc00472 expression compared with empty vector. To study the effect of Linc00472 on CRC cell proliferation, CCK-8 assay was performed in HT-29 and HCT-116 cells. Elevated Linc00472 expression evidently suppressed HT-29 and HCT-116 cell proliferation (Figure 2B and 2C). Moreover, colony formation assay suggested that Linc00472 overexpression obviously reduced colony number in HT-29 and HCT-116 cells (Figure 2D and 2E). All these results indicated that ectopic Linc00472 expression contributed to CRC cell proliferation inhibition. To further explore the effect of Linc00472 on CRC cell apoptosis, flow cytometry analysis was carried out in HT-29 and HCT-116 cells. Up-regulated Linc00472 led to apoptosis induction in HT-29 and HCT-116 cells (Figure 2F and 2G). Taken together, Linc00472 could suppress proliferation and induce apoptosis in CRC cells.

### Linc00472 was a miR-196a sponge

**Figure 3:**
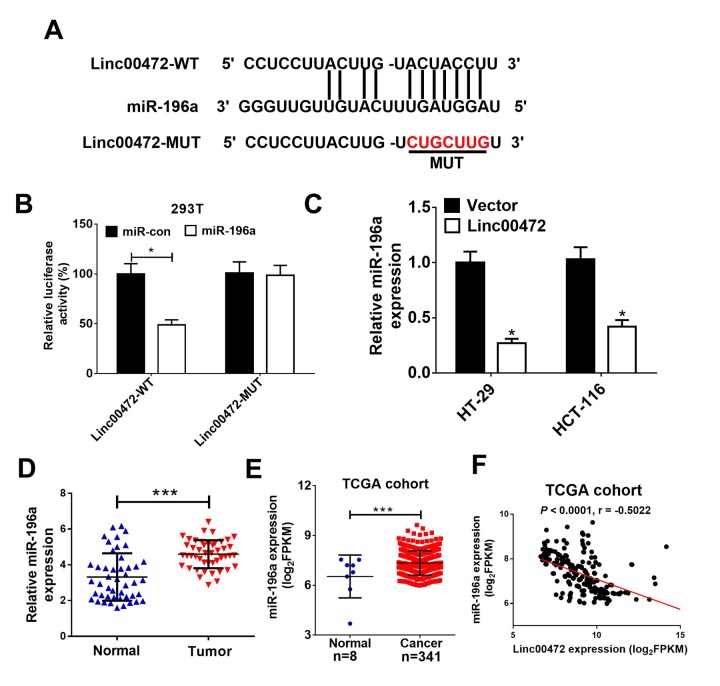
**Linc00472 directly inhibited miR-1496a expression.** (**A**) The predicted binding sites between Linc00472 and miR-141 were shown. (**B**) The luciferase activity was detected in 293T cells. (**C**) qRT-PCR analysis miR-196a expression in HT-29 and HCT-116 cells transfected with pcDNA3.1 vector (Vector) or pcDNA-Linc00472 (Linc00472). (**D**) qRT-PCR assay was performed to detect miR-196a expression in CRC tumor tissues and adjacent normal tissues. (**E**) Linc00472 expression was analyzed in TCGA COAD cohort. (**F**) Correlation analysis between Linc00472 and miR-196a in CRC tissues from TCGA COAD dataset. *P < 0.05, ***P < 0.001.

To further investigate the molecular mechanism of Linc00472 in CRC progression, starBase v2.0 (http://starbase.sysu.edu.cn/) was used to predict Linc00472 targeting miRNAs. As shown in Figure 3A, miR-196a was a predicted target of Linc00472. To validate the direct interaction between mir-196a and Linc00472, the luciferase reporter assay was performed. As expected, the relative luciferase activity of Linc00472-WT was evidently reduced by elevated miR-196a (Figure 3B). However, miR-196a overexpression had no significant effect on luciferase activity of Linc00472-MUT in 293T cells. Moreover, up-regulation of Linc00472 remarkably inhibited miR-196a expression in HT-29 and HCT-116 cells (Figure 3C). Furthermore, miR-196a expression was obviously increased in CRC tissues (Figure 3D), further confirmed by TCGA cohort analysis (Figure 3E). Spearman’s correlation analysis also found that miR-196a expression was inversely associated with Linc00472 level in CRC tissues from TCGA cohort (Figure 3F). All these findings demonstrated that Linc00472 down-regulated miR-196a expression by direct interaction.

### PDCD4 was directly targeted by miR-196a

**Figure 4:**
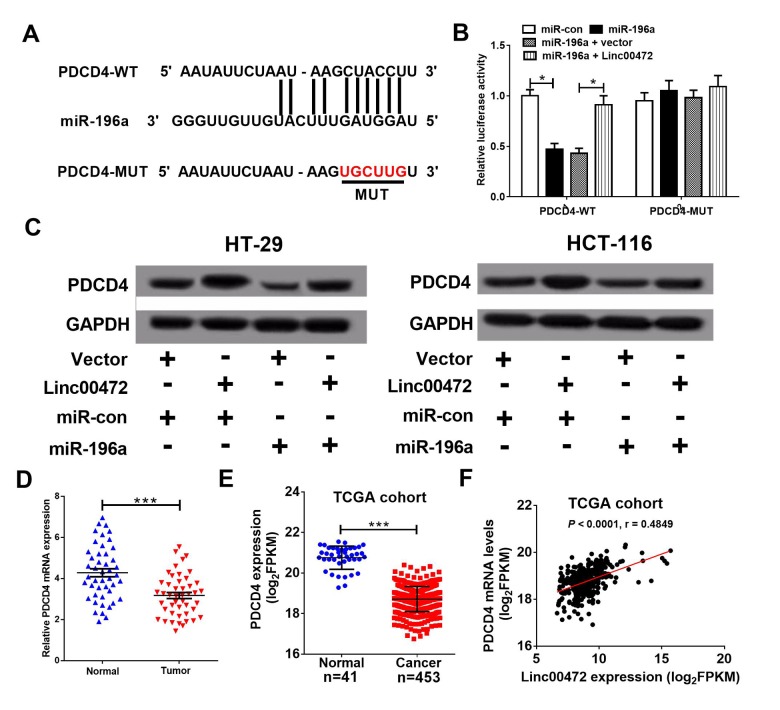
**PDCD4 was a target of miR-196a.** (**A**) The putative binding sites between PDCD4 and miR-196a by miRanda software. (**B**) The luciferase activity of wide-type or mutant-type PDCD4 was determined in 293T cells. (**C**) PDCD4 protein level in HT-29 and HCT-116 cells was detected using western blot analysis. (**D**) qRT-PCR assay analyzed PDCD4 mRNA levels in CRC tumor tissues and adjacent normal tissues. (**E**) PDCD4 mRNA levels were analyzed in TCGA COAD cohort. (**F**) Correlation analysis between Linc00472 and PDCD4 in CRC tissues from TCGA COAD dataset. *P < 0.05, ***P < 0.001.

To further identify target genes of miR-196a, miRanda software was used to predict miR-196a targets. PDCD4 was found to be a potential target of miR-196a (Figure 4A). To further verify the prediction, we performed luciferase reporter assay. Our results indicated that miR-196a overexpression strikingly reduced luciferase activity of wide-type PDCD4 (PDCD4-WT), which was eliminated by elevated Linc00472 in 293T cells (Figure 4B). However, the luciferase activity of mutant type PDCD4 (PDCD4-MUT) had no obvious change among all groups. The result of western blot analysis revealed that Linc00472 promoted PDCD4 expression while miR-196a suppressed PDCD4 expression in HT-29 and HCT-116 cells (Figure 4C). Additionally, Linc00472 could reversed the inhibitory effect of miR-196a on PDCD4 expression. Oppositely, miR-196a overexpression could eliminate the inductive effect of Linc00472 on PDCD4. Furthermore, PDCD4 levels was noticeably decreased in CRC tissues (Figure 4D), further verified by TCGA dataset analysis (Figure 4E). Correlation analysis revealed a positive correlation between PDCD4 and Linc00472 in CRC tissues from TCGA dataset (Figure 4F). These evidences demonstrated that Linc00472 could positively modulate PDCD4 expression by sponging miR-196a.

### Linc00472 inhibited proliferation and enhanced apoptosis by modulating miR-196a/PDCD4 axis in colorectal cells

**Figure 5:**
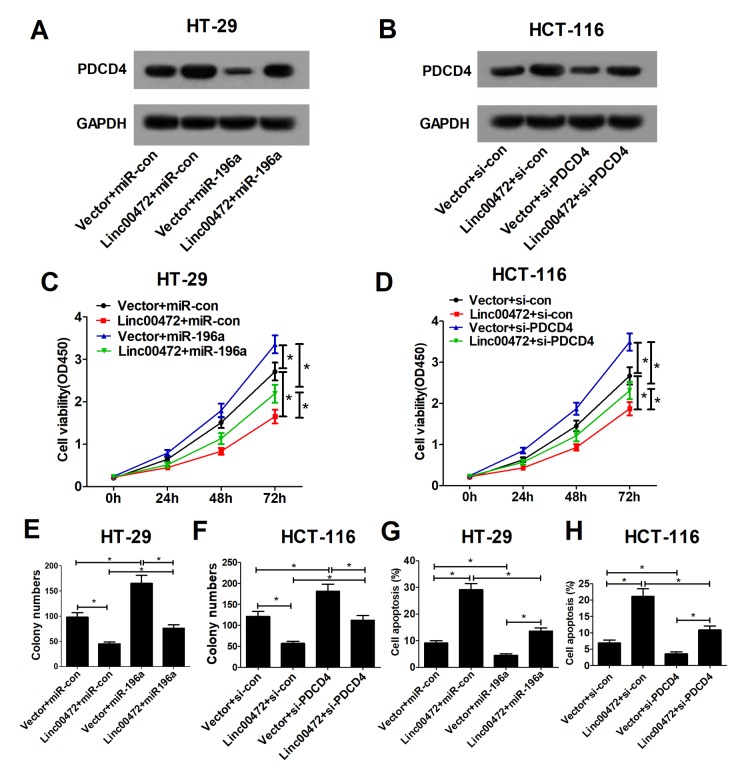
**Linc00472 inhibited proliferation and enhanced apoptosis by modulating miR-196a/PDCD4 axis in CRC cells.** HT-29 cells were transfected with Vector+miR-con, Linc00472+miR-con, Vector+miR-196a or Linc00472+miR-196a, and HCT-116 cells were transfected with Vector+si-con, Linc00472+si-con, Vector+si-PDCD4 or Linc00472+si-PDCD4. The PDCD4 protein levels were measured by western blot assays (**A** and **B**), the cell proliferation was evaluated by CCK-8 assays (**C** and **D**), the cell clone numbers were estimated by colony formation assays (**E** and **F**), and the cell apoptosis was determined with flow cytometry (**G** and **H**). *P < 0.05.

It was further investigated that whether miR-196a or PDCD4 implicated in the effect of Linc00472 on proliferation and apoptosis in CRC cells. As displayed in Figure 5A, Linc00472 could abolish the inhibitory effect of miR-196a on PDCD4 expression. Also, miR-196a also could decline Linc00472-mediated elevation in PDCD4 expression. Additionally, PDCD4 knockdown diminished Linc00472-induced increase in PDCD4 expression (Figure 5B). Function analysis discovered that miR-196a overexpression or PDCD4 knockdown promoted proliferation and induced apoptosis in CRC cells, however, the pro-tumor effect of up-regulated miR-196a or down-regulated PDCD4 was reserved by Linc00472 overexpression. Moreover, miR-196a or si-PDCD4 weakened the anti-tumor effect of Linc00472 on CRC cells (Figure 5C-H). These data recommended that Linc00472 exerted its tumor suppressive role in CRC cells by down-modulating miR-196a and elevating PDCD4.

### Linc00472 suppressed tumor growth *in vivo*

**Figure 6:**
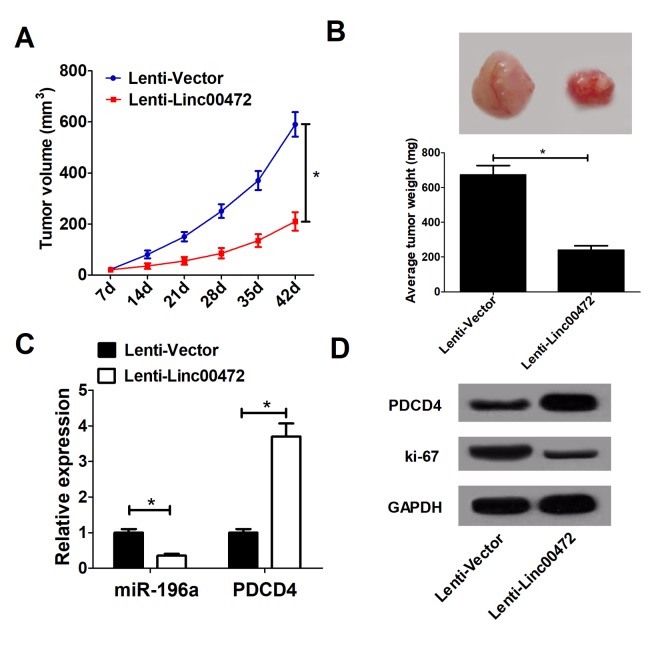
**Linc00472 suppressed tumor growth** ***in vivo*.** (**A**) The tumor volume curve of null mice was analyzed. (**B**) The tumor weight of null mice was measured. (**C**) The miR-196a expression and PDCD4 mRNA levels in tumors of null mice were detected by qRT-PCR. (**D**) Western blot analysis was performed to determine PDCD4 and Ki-67 protein levels. *P < 0.05.

To further explore the effect of Linc00472 on tumor growth *in vivo*, HT-29 cells stably transfected with lenti-Vector or lenti-Linc00472 were subcutaneously injected into nude mice. Linc00472 overexpression hindered tumor growth in nude mice, evidenced by suppressed tumor volume (Figure 6A) and weight (Figure 6B). Moreover, Linc00472 overexpression led to decreased miR-141 expression and increased PDCD4 expression *in vivo* (Figure 6C)*.* Furthermore, Ki-67 protein level was distinctly down-regulated in Linc00472 overexpressing mice tumor tissues (Figure 6D). Collectively, Linc00472 inhibited tumor growth *in vivo*.

## DISCUSSION

Emerging evidence suggested that lncRNAs exerted important roles in various biological processes [17,18]. Growing studies demonstrated that many lncRNAs were aberrantly expressed in cancers and acted as tumor suppressors or oncogenes depending on the anti- or pro-tumor activities of their modulating protein-coding genes [19–21]. In the present study, we found that Linc00472 was significantly down-regulated in CRC tumor tissues compared with normal tissues from our samples and TCGA data. Functional analysis revealed that Linc00472 overexpression inhibited proliferation and enhanced apoptosis *in vitro* and hindered tumor growth *in vivo* in CRC. Moreover, Linc00472 acted as a tumor suppressor by sponging miR-196a and releasing PDCD4. Collectively, our study illuminated a Linc00472/miR-196a/PDCD4 regulatory axis existed in CRC.

Linc00472, a novel lncRNA located on chromosome 6q13, was down-regulated in breast and ovarian cancers [13,14]. Moreover, the Cancer RNA-Seq Nexus database [22] revealed low LINC00472 expression in other cancers, such as lung carcinoma and endometrial carcinoma. LINC00472 overexpression inhibited breast cancer cell proliferation, migration and contributed to better prognosis [23]. All these studies suggested that Linc00472 may act as a tumor suppressor in cancers. However, the expression and function of Linc00472 were little-known in CRC. In the present study, we found that Linc00472 expression was decreased in CRC tissues, further verified by TCGA cohort. In parallel with our study, two latest publications also revealed low Linc00472 expression presented in CRC [15,24]. Our study further demonstrated that Linc00472 suppressed proliferation and induced apoptosis in CRC cells. These findings recommended that Linc00472 could act as a tumor suppressor in CRC.

Accumulating evidence suggests that lncRNAs exert functions by acting as competing endogenous RNAs (ceRNAs) to sponge miRNAs and de-repress their targeting mRNAs. For instance, lncRNA TUG1 promoted CRC cell migration and invasion and EMT-related proteins through acting as a ceRNA to bind with miR-422a and modulate its function [10]. In our study, we investigated the functional mechanism of Linc00472 in CRC cells and discovered that Linc00472 involved in the ceRNA regulatory network and functioned as endogenous miRNA sponges to bind to miR-196a and regulated its function. In line with our finding, Lu et al. [25] reported that ectopic Linc00472 expression inhibited cell proliferation and invasion, facilitated apoptosis and sensitized breast cancer cells to doxorubicin by sponging miR-141. Mounting studies demonstrated that miR-196a functioned as an oncogene or a tumor suppressor in various cancers. For example, miR-196a was increased in gastric cancer (GC) and promoted GC cell proliferation, migration and invasion [26,27]. In contrast, miR-196a expression was reduced in renal cell carcinoma and elevated miR-196a inhibited proliferation and migration and enhanced apoptosis of renal cell carcinoma cells [28]. Moreover, miR-196a was reported to be up-regulated and promoted cell growth and stemness of CRC cells by targeting Zymogen Granule Protein 16 [29]. However, how miR-196a was controlled in CRC remained unclear. In the current work, we found that miR-196a promoted proliferation and suppressed apoptosis in CRC cells, and bioinformatics prediction and luciferase reporter assays confirmed that miR-196a directly bind to Linc00472. More importantly, miR-196a could inverse the tumor suppressive effects of Linc00472 in CRC, suggesting Linc00472 suppressed CRC progression, at least in part, through directly inhibiting miR-196a.

PDCD4 has been demonstrated to be dramatically down-regulated and act as a tumor suppressor in various cancers [30–32]. For instance, elevated PDCD4 expression inhibited tumor growth and promoted apoptosis in GC [30]. In addition, previous studies revealed that PDCD4 inhibited proliferation and invasion and induced apoptosis in CRC cells [33,34]. Although the functional role of PDCD4 was well known, how PDCD4 expression was regulated during tumorigenesis required more researches to elucidate. Our study further demonstrated that PDCD4 was down-regulated in CRC tissues and directly targeted by miR-196a, which confirmed previous prediction [35]. Linc00472 overexpression alleviated the inhibitory effect of miR-196a on PDCD4 expression. Furthermore, PDCD4 knockdown abolished the tumor suppressive effects of Linc00472 in CRC cells. Those effects could be partially attributed to Linc00472 functioning as a ceRNA for miR-196a. However, how Linc00472 is regulated, the controlled downstream signal pathway and the clinical value of this study needs more comprehensive and deep-going research.

In conclusion, our study revealed that Linc00472 was down-regulated in CRC tissues and cells and elevated Linc00472 expression suppressed proliferation and promoted apoptosis via miR-196a/PDCD4 axis. Our study suggested that Linc00472 may serve as an effective therapeutic target for CRC.

## MATERIALS AND METHODS

### Tissues specimens and cell culture

Forty-six pairs of CRC tumor and adjacent non-tumor tissue samples were collected from the First Affiliated Hospital of Zhengzhou University. Written informed consent was obtained from all patients. This study was performed with the approval of Ethics Committee of the First Affiliated Hospital of Zhengzhou University. The normalized RNA-seq data of colon Adenocarcinoma (COAD) were downloaded from the TCGA data portal website (https://cancergenome.nih.gov/).

The immortalized human colonic epithelial cell line (NCM460), four human CRC cell lines (SW480, SW620, HT-29 and HCT-116) and embryonic kidney 293T cell line (293T) were obtained from American Tissue Culture Collection (ATCC, Manassas, VA, USA). All cells were cultured in DMEM (Gibco, Carlsbad, CA, USA) supplemented with 10% FBS (Invitrogen, Carlsbad, CA, USA) at 37°C, 5% CO_2_ conditions.

### Transfection

The Linc00472-overexpressing pcDNA-Linc00472 plasmid (Linc00472) and empty pcDNA3.1 vector (Vector), miR-196a mimic (miR-196a) and scramble control (miR-con), and PDCD4 small interference RNA (si-PDCD4) and its scramble control (si-con) were synthesized from GenePharma (Shanghai, China). Cell transfection was carried out using lipofectamine 2000 reagent (Invitrogen) according to the protocol provided by the manufacturer.

### Quantitative real-time PCR (qRT-PCR) assays

Total RNA extraction from CRC tissues and cells was performed with TRIzol reagent (Invitrogen). Quantitative real-time PCR (qRT-PCR) was carried out using SYBR Green Realtime PCR Master Mix (TaKaRa, Dalian, China) on a CFX96 Real-time PCR Detection System (Bio-Rad, Hercules, CA, USA). Linc00472 and PDCD4 expression levels were normalized to GAPDH expression while miR-196a was normalized to U6 level, respectively. In addition, melting curves were used to evaluate to amplification specificity. Data were analyzed using the comparative Ct method (2^−△△Ct^) [36].

### Western blot assays

Total protein was extracted using Radio-Immunoprecipitation Assay buffer (Thermo Scientific, Rockford, IL, USA). Then protein samples were separated on SDS-PAGE and transferred onto nitrocellulose membrane (Bio-Rad). Primary antibodies anti-PDCD4, anti-Ki-67 and anti-GAPDH were obtained from Santa Cruz Biotechnology (Santa Cruz, CA. USA). The protein bands were detected using the BeyoECL Plus reagent (Beyotime Institute of Biotechnology, Jiangsu, China).

### Luciferase reporter assays

The wild or mutant Linc00472 and PDCD4 3’ UTR containing predicted miR-196a binding sites were amplified and integrated into pGL3 vector to generate wild type pGL3-Linc00472 (Linc00472-WT) and pGL3-PDCD4 (PDCD4-WT) or mutant type pGL3-Linc00472 (Linc00472-MUT) and pGL3-PDCD4 (PDCD4-MUT) vectors. Then the constructed vectors were respectively co-transfected into 293T cells with miR-con or miR-196a, along with Vector or Linc00472. At 48 h post transfection, Dual-Luciferase Reporter Assay System (Promega) was used to determine the luciferase activity.

### Cell viability and colony formation assays

The cell proliferation of HT-29 and HCT-116 was assessed by Cell Counting Kit-8 (CCK-8) assay (Beyotime, Shanghai, China). Cells were plated into the 96-well plate and cultured for 0, 24, 48, and 72 h. The absorbance at 450 nm was detected by a microplate reader. For colony formation assay, HT-29 and HCT-116 cells were seeded into 6-well plates and cultured for two weeks. Then, the cell colonies were stained with crystal violet and counted.

### Flow cytometry

HT-29 and HCT-116 cells were collected and stained with 5 μl Annexin V/FITC and 5 μl PI for 15 minutes in darkness at room temperature. Then, cell apoptosis was determined using flow cytometry analysis (BD Biosciences, San Jose, CA, USA).

### Tumor formation in nude mice

HT-29 cells stably transfected with lentivirus vector of overexpressed Linc00472 (Lenti-Linc00472) or negative control (Lenti-Vector) were subcutaneously injected rear flank of nude mice (6 per group). The tumor volumes were measured with calipers every 7 day. At 42 day after injection, mice were sacrificed, and tumor weight was measured. All animal procedures were performed with the approval of the Local Medical Experimental Animal Care Commission.

### Statistical analysis

All data present as mean ± standard deviation (mean ± SD) from at least three independent experiments. All analyses were carried out using GraphPad Prism 7 software or SPSS 20.0 software. Overall survival (OS) curve was performed by Kaplan-Meier analysis. The Student’s t-test was used to evaluate the difference between two groups. One-way ANOVA analysis was implemented for multiple group comparisons. *P < 0.05* was considered statistically significant.
